# Factors Associated with the Implementation of Pediatric Immunization Services: A Survey of Community Pharmacies

**DOI:** 10.3390/vaccines12010093

**Published:** 2024-01-18

**Authors:** Oluchukwu M. Ezeala, Nicholas P. McCormick, Christopher L. Meininger, Spencer H. Durham, Tessa J. Hastings, Salisa C. Westrick

**Affiliations:** 1 Department of Health Outcomes Research and Policy, Harrison College of Pharmacy, Auburn University, Auburn, AL 36849, USAnzm0066@auburn.edu (N.P.M.); clm0095@auburn.edu (C.L.M.); 2 Department of Pharmacy Practice, Harrison College of Pharmacy, Auburn University, Auburn, AL 36849, USA; durhash@auburn.edu; 3 Department of Clinical Pharmacy and Outcomes Sciences, College of Pharmacy, University of South Carolina, Columbia, SC 29208, USA; hastint@mailbox.sc.edu

**Keywords:** pediatric, immunization, vaccines, PREP Act, pharmacies, pharmacists, community, barriers, roles, vaccination

## Abstract

Pharmacists are well-positioned to help increase pediatric immunization rates. This study assessed the types of pediatric vaccines offered in community pharmacies, compared participant/pharmacy characteristics and participants’ perceptions of barriers and pharmacists’ role in providing pediatric immunizations between pharmacy-based providers and non-providers, and assessed factors associated with pharmacy-based pediatric immunization provision. A cross-sectional survey was sent to Alabama community pharmacies from February to April 2023, of which 240 responded (20.5% response rate). Measures included whether they offered childhood vaccines in 2022 and the types of vaccines administered, participants’ perceptions of pharmacists’ role in pediatric immunization, and perceived barriers to providing pharmacy-based pediatric immunizations. Roughly half of pharmacies (50.8%) provided pediatric immunization services with influenza vaccines (91.0%) the most commonly provided vaccines and poliovirus-inactivated vaccines (4.9%) the least. Pharmacies providing pediatric immunization services significantly differed from non-providers. That is, the majority of providers practiced within a grocery or retail store; they were younger and practiced in a pharmacy with higher average daily prescription volume and a higher average pharmacy practice full-time equivalent; and they perceived lower implementation logistics barriers and a lower role of pharmacists regarding pediatric immunization. Multivariable logistic regression analysis indicated that implementation logistics is significantly associated with pharmacies offering pediatric immunization services after controlling for pharmacy/participant characteristics (*p* = 0.01). Therefore, ameliorating implementation logistics barriers should be considered when devising strategies to promote pediatric immunization services in community pharmacies.

## 1. Introduction

Pediatric immunization offers a safe and effective means of protecting children against potentially fatal vaccine-preventable diseases [1]. Achieving optimal immunization rates among this population is essential to prevent infectious disease outbreaks and protect against serious sequelae from infection [2]. Following the declaration of the COVID-19 pandemic as a national emergency by the US president on 13 March 2020 [3], various states, including Alabama, adopted safety measures such as social distancing and lockdowns to combat the disease [4,5,6]. Consequently, there was a decline in pediatric immunization rates which may be partially attributed to parents’ avoidance of well-child visits for fear of exposing their children to the virus [5,6,7]. According to the Centers for Disease Control and Prevention (CDC), the Vaccines for Children (VFC) program recorded a significant reduction in healthcare providers’ requests for VFC-covered non-influenza vaccines recommended by the CDC’s Advisory Committee on Immunization practices (ACIP) for children. This reduction occurred between March and April 2020 when compared to the same period in 2019 [6]. Similarly, in the state of Alabama, the immunization rate for pediatric vaccines including measles, mumps, and rubella (MMR); varicella and hepatitis B decreased notably by approximately 51% to 59% from March to May 2020 compared to March to May 2019 [5]. Although the numbers improved among children aged 24 months and older, as movement restrictions were lifted and daily hospital activities gradually returned to normal, they still remained relatively lower than pre-pandemic levels [5].

To address this problem, the US Department of Health and Human Services (HHS), in August 2020, acknowledged the significant role of pharmacists in enhancing access to childhood immunizations [8]. HHS granted approval across all 50 states, superseding state laws, through a third amendment to the declaration under the Public Readiness and Emergency Preparedness (PREP) Act for licensed pharmacists to both request and administer US Food and Drug Administration-approved vaccines recommended by ACIP, including COVID-19 and seasonal influenza vaccines, to individuals aged 3 to 18 years [8]. Prior to the PREP Act, state-level restrictions for pharmacists varied in terms of the type of vaccines permitted to administer, minimum age requirements for vaccine recipients, and protocols or prescription requirements [9,10]. For example, Alabama’s pharmacy practice statutes allow pharmacists to administer any vaccine to individuals of any age under approved protocol [10], whereas South Carolina pharmacy laws require pharmacists to obtain a prescription before administering any vaccine, with the exception of influenza, to individuals younger than 18 [10]. As a result of the PREP Act, 12–13% more influenza immunizations were given to children at pharmacies over the 2020 and 2021 flu periods when compared to the 2018 and 2019 (7–10%) influenza periods [11]. However, with the recent eleventh amendment to the PREP Act declaration, this authorization has ended for routine childhood vaccines but remains in effect for COVID-19 and influenza vaccines until 31 December 2024 [12]. This will be the case unless further extension or permanent adoption through state laws occurs, thereby enabling pharmacists to play a more active role in pediatric vaccine administration [8,13].

Even though pediatric immunization rates have improved from the pandemic period, these rates still fall below the desired level. In the case of influenza immunization, the CDC reported that compared to the 2020/2021 flu season, the coverage rate for children aged 6–17 years dropped by 0.8% in 2021/2022 [14]. Additionally, Seither and colleagues found that the nationwide percentage of kindergarten children who received the state-mandated immunizations fell from roughly 94% in the 2020/2021 school year to 93% in the 2021/2022 school year [15]. Previous studies have consistently recognized pediatrician offices as the primary location for pediatric immunizations because of reasons such as concerns about follow-up care [16]. For example, Gates and colleagues reported that approximately 95.0% of pediatric influenza immunizations were administered in pediatrician offices with only 5% administered in pharmacies before the PREP Act was implemented [17]. Various barriers such as regulatory restrictions, reimbursement issues, and lack of physician and parental buy-in have also been historically identified as contributing factors to why pharmacists are not participating to their full potential [16,17]. However, little is known regarding the types of pediatric vaccines being offered and factors facilitating the implementation of pediatric immunization services in community pharmacies in Alabama following the PREP Act. Therefore, the objectives of this study were to (1) assess the extent of community pharmacies providing pediatric immunization, (2) compare pediatric immunization providers and non-providers in terms of their characteristics, perceived barriers, and perceived roles as immunizers, and (3) assess factors associated with the provision of pediatric immunization services when controlling for participant/pharmacy characteristics. Realization of these objectives would provide valuable insights when designing specific interventions aimed at enhancing pediatric immunization services in community pharmacies.

## 2. Materials and Methods

### 2.1. Study Population

This cross-sectional study utilized a self-administered mail and online survey (Questionnaire, Supplementary Material (S1)) of community pharmacies in Alabama. Alabama was chosen because a substantial decline in immunization rates across various vaccines among children was observed during the COVID-19 pandemic [5]. All community pharmacies were recruited using the Hayes Directory, which includes a list of 1172 Alabama community pharmacies [18]. The unit of analysis was at the pharmacy level. One key informant represented each pharmacy and consisted of pharmacy owners, managers, or staff pharmacists. All procedures were approved by the first author’s Institutional Review Board as an expedited review.

### 2.2. Data Collection

A mixed-mode survey (paper and electronic format) was distributed from February to April 2023. Up to 5 contacts were made. Five contacts, addressed to the pharmacy manager, were used, including a pre-notification postcard, a survey packet, a reminder postcard, a fax (or a letter for those with no fax information), and a replacement survey packet; all were delivered via postal services. The pharmacy manager could participate in the survey or pass the questionnaire to another pharmacist who was more knowledgeable about the immunization service at that pharmacy. A QR code was provided on each contacting medium that led to an online version of the survey for those who preferred to complete the survey electronically. To ensure that multiple pharmacists from one location did not complete the survey, a unique 4-digit identifier was assigned to each pharmacy, which was also required to access the electronic survey. Pharmacists who completed the survey were offered a $25 e-gift card for their time.

### 2.3. Measures

First, pediatric immunization practices in community pharmacies were assessed by asking whether their practice site provided immunization services to children aged 10 or younger in 2022. Those who offered immunization services to the population of interest were then asked to indicate the types of pediatric vaccines administered in in the same year, 2022, e.g., COVID-19, influenza, and varicella. Second, 17 items were used to measure the pediatric immunization barriers (PI-Bs) with 4-point Likert response categories ranging from 1 = ‘not a barrier’ to 4 = ‘major barrier’. This PI-B measure was developed using two pre-existing measures by Hastings et al. and Islam et al. Of the 17 items, 10 items were from Hastings et al.’s measure to assess barriers to pharmacy-based pediatric immunization services [19]. These comprise items 2–7, 10, and 13–15 of our PI-B measure. An additional seven items from Islam et al. assessed challenges to vaccine implementation to adolescents and adults for pharmacists [20]. These items were then modified from an adult and adolescent immunization context and closed-ended, check-if-apply questions to a pediatric immunization context, and a 4-point Likert scale to match with the other 10 items to generate items 1, 8, 9, 11, 12, 16, and 17 of the PI-B measure. Third, a 5-item, 4-point Likert scale ranging from 1 = ‘strongly agree’ to 4 = ‘strongly disagree’ focusing on pharmacists’ perceived pediatric immunization roles (PI-R) was adapted from a measurement assessing pharmacist perceived roles in the delivery of adult vaccines in an unpublished GlaxoSmithKline (GSK, Philadelphia, PA, USA) sponsored study. Other measures assessing pharmacist and pharmacy characteristics were obtained from previous research studies [21,22,23].

### 2.4. Data Analysis

Potential non-response bias was investigated by comparing the first 15% to the last 15% of the responders in terms of their characteristics using Chi-square and one-way ANOVA tests. To ensure that no duplicate responses occurred for the same pharmacy, we only included the first response and eliminated subsequent responses from the final data set. As a result, we removed multiple mail-in surveys (*n* = 6) and duplicate online survey responses (*n* = 19). Furthermore, surveys with less than 61% complete (*n* = 4) were also removed. In relation to the handling of the missing data, 4 pharmacists had completely missing data for the PI-B and PI-R measures due to skipping the page on the mail-in form. When analyzing the PI-B and PI-R measures, these individuals were excluded on a case-wise basis as they had no values to contribute to the EFA(s) and logistic regression model generation. Outside of the PI-B and PI-R measures, individuals were excluded from analyses on a case-wise basis if they had any missing data in the variables of importance; i.e., responses were excluded from the logistic regression model if they were missing any of the included demographic, PI-B subscale, or PI-R scale predictors or the response variable. A total of 9 pharmacists were excluded from the logistic regression model, utilizing a final total of 231 when generating the model.

Participant characteristics, pharmacy characteristics, and pharmacy involvement in pediatric immunization practices were analyzed using descriptive statistics. Exploratory factor analysis (EFA) was conducted on the PI-B measure using a maximum likelihood extraction method and varimax rotation with Kaiser normalization to assess the presence and validity of constructs within the measure. Components with eigenvalues ≥1.0, were retained and scale items with factor loadings <0.400 were dropped from analysis [24,25]. The EFA results are shown in Table S1. In the initial EFA of the PI-B measure, three items were identified for removal due to their failure to load. These excluded items are as follows: Item (8)—“reimbursement/insurance issues”, Item (9)—“time constraints of pharmacy personnel”, and Item (17)—“caregiver afraid/uncooperative/opposed”. Following the item reduction, a subsequent EFA of the PI-B measure identified four components: pediatric immunization knowledge, pediatric immunization proficiency and attitude, implementation logistics, and child vaccine apprehension. The appropriateness of factor analysis was assessed utilizing the Kaiser–Meyer–Olkin Measure of Sampling Adequacy (KMO) and Bartlett’s Test of Sphericity. The appropriateness for factor analysis was determined to be adequate with a KMO = 0.803 and Bartlett’s Test of Sphericity: ×2 (*n* = 92) = 1678.61 (*p* < 0.001). Next, internal consistency of the four PI-B components was assessed using Cronbach’s alpha detailing acceptable internal consistency (α ≥ 0.700). Similarly, exploratory factor analysis (EFA) was conducted on the PI-R measure and resulted in the extraction of a single component. Next, the internal consistency of the PI-R was also assessed determining the drop of items 3 and 5 to reach a valid Cronbach’s alpha ≥ 0.700. Table S2, in Supplementary Material, summarizes characteristics of the PI-B (4 components) and the PI-R measures in terms of the number of items, the scale reliability, and the scale means ± standard deviation (SD).

Bivariate analyses (Chi-square, Mann–Whitney U and one-way ANOVA tests) were conducted to compare providers and non-providers of pediatric immunization services in terms of participant characteristics, perceived barriers (using components from PI-B), and perceived roles (using PI-R). A final multivariable logistic regression model was established utilizing significant factors identified in the bivariate analyses at an a priori alpha level of 0.05. All analyses were conducted using SPSS Statistical Software version 29 (SPSS Inc., Chicago, IL, USA).

## 3. Results

The survey was completed by 240 pharmacies out of the 1172 pharmacies on the mailing list, resulting in a response rate of 20.5%. With a confidence level of 95%, the margin of error was estimated to be 6.3%. In terms of potential non-response bias, no differences were identified in the characteristics of early and late respondents.

Table 1 summarizes the participant and pharmacy characteristics. The average age of the participants was 41.8 years (±10.6) with the majority (58.8%) identifying as female. Non-white participants made up a smaller proportion (12.9%), as did Hispanics and Latinos (0.8%). The most common practice location was standalone independent pharmacy (51.3%), while pharmacy within a medical clinic or hospital was the least common (7.9%). A greater percentage (75.8%) of the participants practiced in urban areas. The majority of the participants (78.3%) reported that they were the pharmacist owner at their practice location. Only about a quarter (23.3%) of the participants did not have a PharmD degree. Interestingly, the percentages of pharmacies that provided pediatric immunization services (50.8%) and those that did not (49.2%) were roughly equal. Among the pediatric vaccines provided, influenza was the most common at 91.0%, which was followed by COVID-19 at 69.7%. Fewer than 15% of providing pharmacies offered *Haemophilus influenzae* type b (7.4%), hepatitis A (13.1%), pneumococcal conjugate (10.7%), and poliovirus inactivated (4.9%) vaccines.

When comparing community pharmacies that provided pediatric immunization services to those that did not (Table 2), significant differences were found in various variables, including pharmacy type/setting, pharmacist age, average daily prescription volume, average pharmacy practice full-time equivalents as well as the implementation logistics barrier, and pharmacists’ perceived role. Specifically, most of the providers practiced within a grocery or retail store; they were also younger and practiced in a pharmacy with higher average daily prescription volume and a higher average pharmacy practice full-time equivalent; and they perceived lower implementation logistics barrier and lower pharmacists’ pediatric immunization role.

The multivariable logistics regression analysis results in Table 3 further reveal that only the implementation logistics barrier component (*p* = 0.01) is significantly associated with the odds of providing pediatric immunization services after controlling for pharmacy and participant characteristics. Specifically, one unit increase in implementation logistics barrier brings the odds to 0.55. As such, the related probability of offering pediatric immunization services is 0.55/(1 + 0.55) = 0.35, or 35%.

## 4. Discussion

This study contributes significantly to the existing body of literature by not only describing the types of pediatric vaccines offered in community pharmacies but also by identifying factors associated with community pharmacists’ provision of pediatric immunization services for ACIP-recommended childhood vaccines following the PREP Act. The study’s findings reveal that although community pharmacists in Alabama have been legally authorized to administer vaccines to individuals of all ages prior to the PREP Act [10], only 50.8% of those surveyed reported offering pediatric immunization services in 2022 to children aged 10 and younger. The study’s results further indicate that only a smaller proportion of these community pharmacies provide ACIP-recommended childhood vaccines, such as *Haemophilus influenzae* b (Hib), Tdap, inactivated poliovirus, and pneumococcal conjugate vaccines. Conversely, influenza and COVID-19 vaccines are the most commonly offered pediatric vaccines in community pharmacy settings. These findings are consistent with a recent Washington-based study, where pharmacists are permitted to give immunizations to children ≥ 6 months, which also noted influenza vaccines as the most frequently offered while identifying inactivated poliovirus and Hib vaccines as among the less frequently provided [26]. Future studies should continue to assess community pharmacy involvement in pediatric immunization services after the end of the PREP Act and compare pharmacy involvement among states with varying levels of permissiveness of state laws concerning pharmacist authority to vaccinate children.

Generally, community pharmacists have been identified as the most accessible healthcare providers in the literature [27]. Community pharmacies are usually the most accessible because they often provide walk-in services, allowing patients to receive immunizations without making an appointment [28,29,30]. This convenience, along with shorter wait times and strategic community-based locations, reduces the need for patients to travel long distances to access essential healthcare services [28,29,30]. Our study results show that the pharmacy setting is associated with the odds of providing pediatric immunization services, with a significant proportion— 61.5% of pharmacy providers— being in retail stores including corporately owned pharmacies, mass merchandise pharmacies and grocery stores (see Table 2 and Table 3). These pharmacies are likely to have extended operating hours as well as provide a one-stop shop for the family; as such, providing immunizations for children could be part of their business strategies. In contrast, standalone independent pharmacies and pharmacies within clinics/hospitals may have more restricted hours, potentially closing early or not operating during weekends. Despite these logistic benefits, the existing literature suggests that regarding pediatric immunization services, pediatrician offices remain the most preferred destination [16,17]. Concerns about follow-up, potential issues with missing or incorrect immunization records, parents’ beliefs that pediatrician offices are safer, and a lack of knowledge about pharmacists’ authorization to administer vaccines all contribute to this preference [16,31,32,33]. Strategies to address some of these concerns have been proposed, such as requiring pharmacists to retrieve immunization records from immunization information systems (IIS) before vaccine administration, reporting immunization records to IIS, and referring pediatric patients and their parents/caregivers for well-child visits [8]. Additionally, pharmacists can play an important role in educating pediatric parents about the importance of immunization and their expanded roles in vaccine administration [34], which can help promote immunization services in the state.

Prevailing sentiments throughout the literature examining pediatric immunization service provision in the pharmacy setting describe common difficulties and barriers in service implementation such as physician perceptions [13,16,35], parental attitudes [16,34], pharmacy space [32,36], workflow [16], hurdles due to corporate [26,36], insurance [26], legislative policy [13,26], and patient documentation accessibility [13,36,37]. Strikingly, the EFA of our internally validated PI-B measure assessing pharmacists’ perceived barriers in pediatric immunization delivery inherently collated these individual items in its implementation logistics component. This lends support to the relationship between these barriers and, consequently, the ability to reliably measure pharmacists’ perceptions of them concurrently and systematically. Bivariate analysis identified the implementation logistics as a significant factor associated with providing pediatric immunization services. The implementation logistics component’s position as a significant factor associated with pharmacists’ pediatric immunization service provision is further supported in our resulting multivariable logistic model; however, its relevance and importance cannot be understated as the component resulted as the only significant, non-controlled factor in our regression model. Contextually, the implementation logistics component within our regression model is identified as being negatively associated with pediatric immunization provision, and the related probability of offering pediatric immunization services is 35%. To capitalize on this revelation, the PI-B and, specifically, the validated 6-item implementation logistics component can be further utilized in pharmacy settings to assess the extent to which pharmacists experience this significant barrier. Insights drawn from both this study’s findings and the future, generalized use of PI-B’s implementation logistics component can inform policymakers and stakeholders on the development of targeted interventions to increase the provision of pediatric immunizations in the pharmacy setting.

There are some limitations to this study. First, the study’s data were obtained solely from community pharmacies in Alabama and thus may not accurately represent what is available in other states or outside the United States. Second, the survey also relied on self-reported responses, which introduces the possibility of recall bias and/or social desirability bias. There is also the possibility of self-selection bias, as pharmacists who are more interested or actively involved in pediatric immunization may have responded to the survey more than pharmacists who are not. This can result in a higher proportion of pharmacists with favorable perceptions or practices toward pediatric immunization than there actually is. However, the non-response bias investigation showed that there was no difference in the characteristics of early and late respondents with regard to potential nonresponse bias. Another limitation lies in our operationalization of the types of pediatric vaccines provided, which we treated as a binary variable rather than a continuous one, such as doses administered; this choice might have yielded different results. Furthermore, there were some data collection issues, such as the receipt of duplicate mail-in surveys or instances where respondents completed both mailed and online surveys. We took measures to remove such duplicates, ensuring that each community pharmacy provided only one response. Next, our study did not include all possible variables that could affect pharmacy decisions to be pediatric immunization providers, including general attitudes toward immunizations and vaccine misconceptions. Finally, it is important to note that pharmacists’ perceptions of their barriers and roles may not align precisely with the actual challenges they face in clinical practice or their practical roles.

## 5. Conclusions

This study highlights that influenza and COVID-19 vaccines are the most commonly provided vaccines by community pharmacies in Alabama for children aged 10 or younger, while other pediatric vaccines are less frequently offered. Approximately 51% of surveyed Alabama community pharmacies offered pediatric vaccines in 2022. This study further reveals differences between pharmacies providing pediatric vaccines and those that do not. That is, the majority of providers practiced within a grocery or retail store; they were younger and practiced in a pharmacy with higher average daily prescription volume and a higher average pharmacy practice full-time equivalent; and they perceived lower implementation logistics barriers and lower pharmacists’ pediatric immunization roles. While future investigations may consider these factors when developing interventions to promote pediatric immunization services in community pharmacies, the multivariable logistic regression reveals the importance of addressing the implementation logistics barrier as a key factor. These insights can inform policymakers and healthcare stakeholders in crafting targeted interventions to expand the availability of pediatric immunization services within community pharmacy settings. Future research endeavors might consider replicating this study in a nationally representative sample to gain a more comprehensive understanding of the factors influencing the provision of pediatric vaccines in community pharmacies.

## Figures and Tables

**Table 1:** Participant and pharmacy characteristics, *n* = 240.

Variable	*n* (%)
**Sex**	
*Male*	97 (40.4)
*Female*	141 (58.8)
*Missing*	2 (0.8)
**Race**	
*White*	207 (86.3)
*Non-White or Part Non-White*	31 (12.9)
*Missing*	2 (0.8)
**Hispanic or Latino**	
*Yes*	2 (0.8)
*No*	236 (98.3)
*Missing*	2 (0.8)
**Title at Pharmacy**	
*Pharmacist owner*	188 (78.3)
*Staff pharmacist, non-pharmacist owner/partner/manager, and other*	51 (21.3)
*Missing*	1 (0.4)
**Education**	
*PharmD obtained*	184 (76.7)
*No PharmD obtained*	56 (23.3)
**Pharmacy Type/Setting**	
*Pharmacy within a grocery or retail store*	98 (40.8)
*Standalone independent pharmacy and pharmacy embedded within a medical clinic or a hospital*	142 (59.2)
**Pharmacy Provides Pediatric Immunization Services**	
*Yes*	122 (50.8)
*No*	118 (49.2)
**Pediatric Vaccines Administered in 2022 among Pharmacies Providing Pediatric Immunization Services (Select All That Apply)**	
*Influenza*	111 (91.0)
*COVID-19*	85 (69.7)
*Tetanus contained vaccines (DTaP, Tdap)*	32 (26.2)
*Measles, Mumps, and Rubella (MMR)*	30 (24.6)
*Varicella*	21 (17.2)
*Hepatitis B (HepB)*	19 (15.6)
*Hepatitis A (HepA)*	16 (13.1)
*Pneumococcal conjugate (PCV13)*	13 (10.7)
*Haemophiles influenzae type b (Hib)*	9 (7.4)
*Poliovirus, inactivated*	6 (4.9)
**Pharmacy Rural–Urban Designation ^a^**	
*Urban (RUCA ≤ 3)*	182 (75.8)
*Non-urban (RUCA ≥ 4)*	55 (22.9)
*Missing*	3 (1.3)
	**µ (SD)**
Pharmacist age	41.8 (10.6)
Pharmacy practice FTEs (40 h/wk)	1.9 (0.8)
	**Median (P25, P75) ^b^**
Average pharmacy prescription volume per day	250.0 (150.0, 350.0)

^a^ Rural–Urban Commuting Area Codes (RUCA) are used to determine the degree of rurality. ^b^ 25th and 75th percentile.

**Table 2:** Comparison of pharmacist and pharmacy characteristics, perceived barriers and perceived roles between pediatric immunization providers and non-providers.

	Pediatric Immunization Service		
	Non-Provider	Provider	χ^2^
	*n* = 118 (49.2%)	*n* = 122	
		(50.8%)	
	*n* (%)	*n* (%)	
**Sex**			
*Male*	43 (36.8)	54 (44.6)	1.53
*Female*	74 (63.2)	67 (55.4)	
**Race**			
*White*	105 (89.7)	102 (84.3)	1.56
*Non-White or Part Non-White*	12 (10.3)	19 (15.7)	
**Title at Pharmacy**			
*Owner*	89 (75.4)	99 (81.8)	1.46
*Non-owner*	29 (24.6)	22 (18.2)	
**Education**			
*PharmD obtained*	88 (74.6)	96 (78.7)	0.57
*No PharmD obtained*	30 (25.4)	26 (21.3)	
**Pharmacy Type/Setting**			
*Pharmacy within a grocery or retail store*	23 (19.5)	75 (61.5)	43.76 **
*Standalone independent pharmacy and pharmacy embedded within a medical clinic or a hospital*	95 (80.5)	47 (38.5)	
**Pharmacy Rural–Urban Designation ^a^**			
*Urban (RUCA ≤ 3)*	83 (45.6)	99 (54.4)	3.50
*Non-urban (RUCA ≥ 4)*	33 (60.0)	22 (40.0)	
	**Median (P25, P75) ^b^**	**Median (P25, P75) ^b^**	MWU ^c^
Average pharmacy prescription volume per day	200.0 (125.0–300.0)	277.5 (200.0–385.0)	4898.50 ***
	**µ (SD)**	**µ (SD)**	*F*
Pharmacist Age	43.3 (10.8)	40.3 (10.2)	5.04 *
Pharmacy practice FTEs (40 h/wk)	1.8 (0.8)	2.0 (0.8)	5.65 *
**Perceived Pediatric Immunization Barriers (PI-B) ^d^**			
*Pediatric Immunization Knowledge*	2.1 (0.8)	2.2 (0.8)	0.77
*Pediatric Immunization Proficiency and Attitude*	2.3 (0.8)	2.2 (0.8)	0.16
*Implementation Logistics*	2.1 (0.7)	1.8 (0.6)	14.94 **
*Child Vaccine Apprehension*	2.9 (1.0)	3.1 (0.9)	2.55
**Perceived Pediatric Immunization Role**			
**(PI-R) ^e^**	2.1(0.5)	1.9 (0.5)	7.63 **

* Significant result at *p* ≤ 0.05; ** Significant result at *p* ≤ 0.01; *** Significant result at *p* ≤ 0.001; ^a^ Rural–Urban Commuting Area Codes (RUCA) are used to determine the degree of rurality; ^b^ 25th and 75th percentile; ^c^ Mann–Whitney U-test; ^d^ Responses range from not a barrier (1) to a major barrier (4); ^e^ Responses range from strongly agree (1) to strongly disagree (4).

**Table 3:** Multivariable logistic regression analysis of factors associated with the provision of pediatric immunization services.

Factor	β	S.E.	Wald	Exp (β)	95% CI		*p*-Value
					Lower	Upper	
Pharmacy Type/Setting ^a^	1.55	0.33	22.42	4.70	2.48	8.92	<0.001 **
Pharmacist Age	−0.00	0.02	0.08	1.00	0.97	1.03	0.77
Average Pharmacy Prescription Volume per Day	0.00	0.00	0.50	1.00	0.99	1.00	0.48
Pharmacy Practice FTEs	0.26	0.21	1.44	1.29	0.85	1.97	0.23
Implementation Logistics	−0.60	0.24	6.09	0.55	0.34	0.88	0.01 **
Pharmacists’ Perceived Pediatric Immunization Role	−0.19	0.21	0.86	0.83	0.55	1.24	0.35
Constant	0.53	0.94	0.32	1.70			0.57

^a^ The reference category is standalone independent pharmacy and pharmacy embedded within a medical clinic or a hospital. ** Significant result at *p* ≤ 0.01.

## Data Availability

The data presented in this study are available on request from the corresponding author. The data are not publicly available due to potential breaches of confidentiality, particularly in areas with few pharmacies, and the possibility that openly sharing the data would jeopardize our ability to conduct future research using the same dataset.

## Supplementary Materials

The following supporting information can be downloaded at https://www.mdpi.com/article/10.3390/vaccines12010093/s1, Questionnaire S1: Pharmacy-Based Pediatric Immunization Services Questionnaire; Table S1: Pharmacists’ Perceived Pediatric Immunization Barriers (PI-B) Exploratory Factor Analysis (EFA) at Factor Loading Level (the correlation coefficient for the variable and factor) of 0.40 or greater; Table S2: Pharmacists’ Perceived Barriers and Roles in Pediatric Immunization Services Measures.
