# Supplementary material for: Factors Associated with the Implementation of Pediatric Immunization Services: A Survey of Community Pharmacies
Source: Vaccines (Basel). 2024 Jan 18;12(1):93. doi: 10.3390/vaccines12010093 (PMC10818495; doi:10.3390/vaccines12010093)
Supplement: Supplementary file 1 [file vaccines-12-00093-s001.zip › Vaccines _SM_Table S1.pdf]

**Table S1:** Pharmacists' Perceived Pediatric Immunization Barriers (PI-B) Exploratory Factor Analysis (EFA) at Factor Loading Level of 0.40 or greater†

| | Components | | | |
|---|---|---|---|---|
| Item† | 1 | 2 | 3 | 4 |
| **Pediatric Immunization Knowledge** | | | | |
| *Pharmacists' knowledge of pediatric vaccines* | .853 | | | |
| *Pharmacists' familiarity of pediatric vaccination schedules* | .850 | | | |
| *Screening for pediatric vaccine appropriateness* | .701 | | | |
| **Pediatric Immunization Proficiency and Attitude** | | | | |
| *Pharmacists' experience with pediatric vaccination* | | .854 | | |
| *Pharmacists' pediatric vaccination technique* | | .601 | | |
| *Pharmacists' resistance to vaccinate children* | | .598 | | |
| **Implementation Logistics** | | | | |
| *Physician (e.g., pediatrician) buy-in* | | | .726 | |
| *Legal liability* | | | .631 | |
| *Vaccine prescription requirements* | | | .625 | |
| *Parental buy-in* | | | .584 | |
| *Availability of space for vaccine administration* | | | .483 | |
| *Documentation requirements* | | | .418 | |
| **Child Vaccine Apprehension** | | | | |
| *Children's fear of needles* | | | | .972 |
| *Children afraid/uncooperative/opposed* | | | | .867 |

†Items (8) "reimbursement/insurance issues," (9) "time constraints of pharmacy personnel," and (17) "caregiver afraid/uncooperative/opposed" were dropped due to failing to load at factor loading level < .40. Bartlett's Test of Sphericity: $x^2$(n = 92) = 1678.61 (p < 0.001); Kaiser-Meyer-Olkin MSA = 0.803.
