# Supplementary material for: Factors Associated with the Implementation of Pediatric Immunization Services: A Survey of Community Pharmacies
Source: Vaccines (Basel). 2024 Jan 18;12(1):93. doi: 10.3390/vaccines12010093 (PMC10818495; doi:10.3390/vaccines12010093)
Supplement: Supplementary file 1 [file vaccines-12-00093-s001.zip › Vaccines_ SM_Table S2.pdf]

Table S2: Pharmacists’ Perceived Barriers and Roles in Pediatric Immunization Services Measures

Measure†	Number of items	μ (SD)	Cronbach’s α
Pharmacists’ Perceived Pediatric Immunization Barriers[a]	14	2.21 (0.57)	.855
Pediatric Vaccination Knowledge	3	2.13 (0.81)	.892
Pediatric Vaccination Proficiency and Attitude	3	2.25 (0.82)	.808
Implementation Logistics	6	1.96 (0.68)	.780
Child Vaccine Apprehension	2	2.99 (0.94)	.948
Pharmacists’ Perceived Pediatric Vaccination Role[b]	3	2.29 (0.77)	.747

†Exploratory factor analyses (EFA) of the pharmacists' perceived barrier measure identified 4 components: pediatric vaccination knowledge, pediatric vaccination proficiency and attitude, implementation logistics, and child vaccine apprehension while the EFA of the perceived role measure resulted in only one component.

[a] Responses range from not a barrier (1) to a major barrier (4)

[b] Responses range from strongly agree (1) to strongly disagree (4)
