# Supplementary material for: Factors Associated with the Implementation of Pediatric Immunization Services: A Survey of Community Pharmacies
Source: Vaccines (Basel). 2024 Jan 18;12(1):93. doi: 10.3390/vaccines12010093 (PMC10818495; doi:10.3390/vaccines12010093)
Supplement: Supplementary file 1 [file vaccines-12-00093-s001.zip › Vaccines_SM_Questionnaire S1.pdf]

Pharmacy-Based Pediatric Immunization Services Questionnaire

Section I: Pediatric Immunization Practice

1. Did your practice site provide immunization services to children aged 10 or younger in 2022?
❒ No ➔ Please indicate why your pharmacy did not provide pediatric immunization services __________ Then ➔ Go to Section II
❒ Yes ➔ In what year did your pharmacy first start administering vaccines to children? ________

2. Which of the following vaccines did your pharmacy administer to children aged 10 or younger in 2022? Select all that apply.
❒ COVID-19
❒ *Haemophilus influenzae* type b (Hib)
❒ Hepatitis A (HepA)
❒ Hepatitis B (HepB)
❒ Influenza
❒ Measles, Mumps and Rubella (MMR)
❒ Pneumococcal conjugate (PCV13)
❒ Poliovirus, inactivated
❒ Tetanus containing vaccines (DTaP, Tdap)
❒ Varicella

3. In your pharmacy, what is the youngest age in years that can be vaccinated without a prescription? __________

4. In your pharmacy, what is the youngest age in years that can be vaccinated with a prescription? ________

5. How do you document pediatric immunization records? Select all that apply.
❒ Enter the records into the pharmacy software
❒ Maintain paper records in the pharmacy
❒ Update or issue a patient's personal immunization record
❒ Update immunization records in ImmPrint (immunization registry)
❒ Notify patient's pediatrician/healthcare provider
❒ Other (please explain) ___________

6. How does your pharmacy verify the need for a pediatric vaccine prior to administering? Select all that apply.
❒ Asking parent/guardian
❒ Contacting their primary care provider
❒ Checking the records in pharmacy dispensing software
❒ Checking ImmPRINT (immunization registry)
❒ Other (please explain) __________

7. Does your pharmacy allow co-administration of vaccines for children aged 10 or younger? For example, can you co-administer COVID-19 and influenza immunizations?
❒ Yes
❒ No
❒ Unsure

Section II: Perceptions Regarding Pediatric Immunization

1. Regardless of whether your site provides pediatric immunizations to children aged 10 or younger, please indicate to what extent you foresee each of the following factors as a barrier to the provision of pediatric vaccines in your pharmacy

	Not a barrier	Minor barrier	Moderate barrier	Major barrier
1. Pharmacists' resistance to vaccinate children				
2. Pharmacists' pediatric immunization technique				
3. Pharmacists' experience with pediatric immunization				
4. Pharmacists' familiarity of pediatric immunization schedules				
5. Pharmacists' knowledge of pediatric vaccines				
6. Screening for pediatric vaccine appropriateness				
7. Documentation requirements				
8. Reimbursement/insurance issues				
9. Time constraints of pharmacy personnel				
10. Vaccine prescription requirements				
11. Availability of space for vaccine administration				
12. Legal liability				
13. Physician (e.g., pediatrician) buy-in				
14. Parental buy-in				
15. Children's fear of needles				
16. Children afraid/uncooperative/opposed				
17. Caregivers afraid/uncooperative/opposed				

2. Regardless of whether your site provides pediatric immunizations to children aged 10 or younger, please tell us how strongly you agree or disagree with the following statements about the role of pharmacists in the delivery of vaccines to pediatric patients.

	Strongly Agree	Somewhat Agree	Somewhat Disagree	Strongly Disagree
1. It is my responsibility to see that my pediatric patients receive recommended vaccines, even if they get them somewhere else				
2. Immunizations are a shared responsibility between myself and the other providers my pediatric patient sees				
3. Many of my pediatric patients receive vaccines in medical offices and clinics				

4. It is my responsibility to stock and administer all routinely recommended pediatric vaccines				
5. It is problematic for me to determine what vaccines my pediatric patients need because I do not have access to their medical information				

Section III: Individual and Pharmacy Characteristics

1. Please indicate your sex:
   ❒ Male
   ❒ Female
   ❒ Other. Please specify: ___________

2. Please indicate your race:
   ❒ White
   ❒ Black or African American
   ❒ Asian
   ❒ Native Hawaiian or Other Pacific Islander
   ❒ American Indian or Alaska Native
   ❒ Other. Please specify: __________

3. Please indicate your ethnicity:
   ❒ Hispanic or Latino
   ❒ Not Hispanic or Latino

4. Please indicate your age at your last birthday: _________ years

5. Please indicate your title. Select all that apply.
   ❒ Staff pharmacist
   ❒ Pharmacist pharmacy owner/partner/manager
   ❒ Non-pharmacist pharmacy owner/partner/manager
   ❒ Other. Please specify: __________

6. Please indicate your education/training. Select all that apply.
   ❒ B.S. Pharmacy
   ❒ PharmD
   ❒ Residency in Pharmacy
   ❒ Master of Pharmacy
   ❒ Other. Please specify: __________

7. Which of the following best describes the pharmacy that is your primary practice location?
   ❒ Stand-alone independent pharmacy
   ❒ Pharmacy embedded within a medical clinic or a hospital

❐ Pharmacy within a grocery or retail store
❐ Other. Please specify: __________

8. What is the average prescription volume per day at your primary practice location?
   _____ Prescriptions per day

9. How many FTEs (Full-time equivalents, 40 hrs/wk) of pharmacists, including the pharmacy manager, does your pharmacy schedule, including both PharmD and BS Pharm?
   _____ FTEs

End of the Questionnaire

Incentive Survey

Instructions. Thank you for completing the questionnaire. Please fill in the requested information below in order to receive $25 Amazon gift card. This gift card will be emailed to you.

1. Your name:

2. Your email:

3. Your phone number (in case your email does not work):
